# Discriminative ability of instrumented cognitive-motor assessments to distinguish fallers from non-fallers

**DOI:** 10.1007/s11357-024-01313-x

**Published:** 2024-08-09

**Authors:** Julia Seinsche, Elena Kyprianou, Eling D. de Bruin, Enrico Saibene, Francesco Rizzo, Ilaria Carpinella, Lisa Lutz, Maurizio Ferrarin, Riccardo Villa, Savvina Chrysostomou, Sotiria Moza, Eleftheria Giannouli

**Affiliations:** 1 Department of Health Sciences and Technology, ETH Zurich, Zurich, Switzerland; 2 Department of Health, OST-Eastern Swiss University of Applied Sciences, St. Gallen, Switzerland; 3 Department of Neurobiology, Care Sciences and Society, Karolinska Institutet, Huddinge, Sweden; 4 Istituto di Ricovero e Cura a Carattere Scientifico, Fondazione Don Carlo Gnocchi Onlus, Milan, Italy; 5 Materia Group, Nicosia, Cyprus; 6 Institute of Physiotherapy, ZHAW School of Health Sciences, Winterthur, Switzerland

**Keywords:** Geriatric assessments, Fall risk, Postural sway, Balance, Executive functions

## Abstract

In older populations, sensitive fall risk assessment tools are important to timely intervene and prevent falls. Instrumented assessments have shown to be superior to standardized fall risk assessments such as the Timed Up and Go Test (TUG) and should capture both motor and cognitive functions. Therefore, the aim was to test novel instrumented assessments with and without a cognitive component. One hundred thirty-seven older adults aged 73.1 ± 7.3 years, 38 categorized as fallers and 99 as non-fallers, conducted five instrumented assessments on the Dividat Senso, a pressure sensitive stepping platform, and three standardized geriatric assessments (TUG, TUG-dual task, 30-s Sit-to-Stand Test (STS)). *T*-tests were applied to compare the test performance of fallers versus non-fallers. Furthermore, logistic regression analyses and area under the curve (AUC) analyses were performed. Statistically significant differences between fallers and non-fallers were found in the Go/No-Go test (*p* = .001, *d* = .72), the TUG (*p* = .014, d = .48), and the STS (*p* = .008, *d* = .51). Only the Go/No-Go test contributed significantly to all regression models. Significant AUC values were found for the Reaction Time Test (RTT) (AUC = .628, *p* = .023), Go/No-Go (AUC = .673, *p* = .002), TUG (AUC = .642, *p* = .012), and STS (AUC = .690, *p* = .001). The Go/No-Go test measuring inhibition showed the best discriminative ability suggesting added value of instrumented assessments with a cognitive component for clinical fall risk assessment in relatively healthy older adults. The study should be extended with a frailer population, in which TUG and the other instrumented assessments are possibly good predictors as well.

**Supplementary Information:**

The online version contains supplementary material available at 10.1007/s11357-024-01313-x.

## Background and objectives

Falls are one of the major causes for injuries and injury-related deaths [1] with a global age-standardized mortality rate of 9.2 per 100,000 in 2017 [2]. According to the WHO, the annual incidence of falls in adults older than 65 is 28–35%, escalating to 32–42% in the population above 70 years, indicating an age-related increase in fall risk [1] and resulting in substantial medical costs [3]. To timely intervene at the onset of physical and cognitive deteriorations, which are considered risk factors for falls, assessments capable of detecting various and often subtle functional changes are necessary.

Standardized geriatric assessments, for example, Sit-to-Stand (STS) transition tests [4, 5], the Timed Up and Go Test (TUG) [6–8], the Berg Balance Scale [9], the Tinetti Mobility Test [10], and the Short Physical Performance Battery (SPPB) [11, 12], are commonly employed for fall risk assessment. However, previous research indicates that instrumented versions of those assessments, such as the iTUG and iSTS, are superior in capturing subtle functional changes and thereby in discriminating fallers from non-fallers. This superiority stems from their higher sensitivity in both temporal and spatial parameters [4, 13–15]. Furthermore, instrumented assessments also allow to record parameters beyond mere completion time, encompassing sub movements or acceleration-derived parameters. Such detailed measurements are important, as previous research has revealed that, e.g., specific aspects of the TUG, unobservable with conventional stopwatch-based measurements have added value for identifying fall risk [15].

Another potential limitation of both instrumented and non-instrumented versions of these standardized geriatric assessments is that they primarily focus on motor skills. However, it has been shown that intrinsic risk factors for falls in older adults not only include impaired motor performance, but encompass cognitive impairments as well [16]. Especially the interplay between motor and cognitive functioning is relevant, considering that the vast majority of falls in real life happens during activities that demand the simultaneous execution of both motor and cognitive tasks. Street crossing, for instance, relies on the interaction of motor functions, such as balance and coordination, with cognitive functions, in particular visuospatial attention and executive functions [17–20].

Thus, sensitive instrumented assessment tools which are able to capture both, motor and cognitive functions, are needed [21]. The primary aim of this study was to test whether a novel instrumented assessment battery including assessments with and without cognitive component is able to distinguish between fallers and non-fallers. Additionally, we aimed to assess its added value to predict fall status compared to standardized geriatric assessments that are used to assess fall risk.

## Research design and methods

### Study design

This is a secondary analysis of an international randomized controlled trial (RCT) aiming to investigate feasibility and effectiveness of a 10-week home-based motor-cognitive training program in community-dwelling older adults. The protocol of the RCT has been registered at ClinicalTrials.gov (NCT05751551), and the study design has been described in detail elsewhere [22]. The analyses described below were conducted using pre-intervention data.

### Ethics approval

The study protocol of the RCT was approved by all local ethical committees, including the Cantonal ethics committee in Zurich, Switzerland (2022–01746); the Don Carlo Gnocchi Foundation ethics committee in Italy (06_16/12/2022); and the Cyprus National Bioethics Committee (ΕΕΒΚ ΕΠ 2021 51).

### Participants

In- and exclusion criteria are based on the purpose and requirements of the aforementioned RCT. To be eligible, participants had to meet the following conditions: (1) age of 60 years and older, (2) prescription for rehabilitation (either in an in- or outpatient setting) within the past 6 months, (3) Mini-Mental State Examination (MMSE) score of 24 or higher, (4) physical capability to stand independently for at least 2 min, (5) ability to provide informed consent, and (6) internet access and a TV or PC-screen at home. Exclusion criteria comprised the following: (1) residency in a nursing home, (2) mobility or cognitive limitations or comorbidities that would impair their ability to conduct the intervention and/or the pre-/post-assessments, (3) severe sensory impairments, (4) previous or acute major psychiatric illness (such as schizophrenia, bipolar disorder, recurrent major depression episodes), (5) history of drug or alcohol abuse, (6) terminal illness, (7) participation in another clinical trial, and (8) an expected absence from home of more than 2 weeks during the study period.

Participants who met the inclusion criteria were subsequently randomized to a control group or an intervention group using permuted block randomization. Furthermore, they were classified as either “fallers” or “non-fallers” based on whether they had experienced at least one fall incident within the past 12 months. Thereby, based on the definition of Prevention of Falls Network Europe (ProFaNE), falls were defined as “*an unexpected event in which the participants come to rest on the ground, floor, or lower level*” [23 (p.1619)].

A written informed consent had to be obtained from each participant before any data was collected.

### Materials

**Fig. 1:**
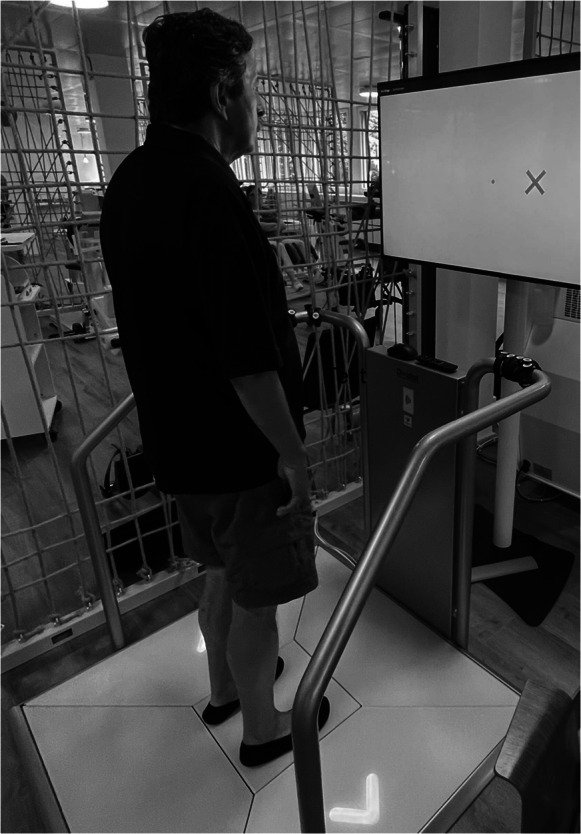
Assessments on the Dividat Senso

The instrumented assessments were conducted on the Dividat Senso (Dividat GmbH, Schindellegi), which is a stepping platform (1.13 m × 1.3 m) consisting of 5 pressure sensitive plates (center, front, back, right, left; each sensor plate has 4 sensors recording at 50 Hz) detecting weight shifting as well as stepping movements. The platform is connected to a screen on which the instructions and stimuli of the assessments appear (Fig. 1).

### Outcomes and outcome measures

#### Instrumented assessments

The assessments were developed using an iterative design process within a focus group study [24] and a usability study [25]. Each instrumented motor-cognitive assessment began with a short practice phase that was not evaluated for the final test’s score. The instrumented assessment battery comprised the following tests:

Instrumented assessments with a cognitive component

1. The Reaction Time Test (RTT) assessing psychomotor speed. In this test, six triangles are presented on the screen, and as soon as a triangle turns dark, participants are instructed to step in the corresponding direction as fast as possible. Outcome measure was the average reaction time (time between stimulus presentation and pressing the platform) across all stimuli.
2. The Go/No-Go Test assessing selective attention and inhibition. In this test, a cross (x) or a plus sign ( +) appear on left or right side of the screen in a randomized order. Participants are instructed to react as fast as possible with a step in the respective direction only when a cross (x) appears. When a plus sign ( +) appears, they are instructed to maintain still. Outcome measure was the average reaction time across all stimuli.
3. The Flexibility Test assessing cognitive flexibility (task switching). In this test, a rounded and an angular figure are presented on the left and right of the screen (in a randomized side order). Participants are instructed to conduct a step towards the rounded figure and then towards the angular figure and so forth in an alternating manner. Outcome measure was the average reaction time across all stimuli.

Instrumented assessments without a cognitive component

(4) The Sway Test assessing postural sway. In this test, participants are instructed to stand still for 30 s. Outcome measures were path length and mean sway speed.
(5) The Coordinated Stability Test assessing dynamic balance. An irregular shape figure is presented on the screen. Participants are asked to track this figure by shifting their center of pressure (COP). Outcome measures were deviations from the ideal path and trace length.

#### Standardized geriatric assessments

The Timed Up and Go (TUG) Test [8] is a measure of mobility and functional balance and required the participant to stand up from a chair, walk 3 m straight ahead, turn, walk back, and sit on the chair again [8]. For the TUG-DT, a secondary cognitive task was added which required participants to count backwards from 90, subtracting serial sevens. Each condition—single and dual task TUG—was performed twice and each time, the time taken for the participant to complete the test was measured. The best (in this case fastest) trials were included in the statistical analysis. Dual-task cost (DTC) was calculated by determining the percentage at which the secondary task interfered with the test performance using the following formula:

DTC [%] = 100 × (DT score − simple task score)/simple task score.

The TUG-DT has shown to be more sensitive to detect age-related declines and risk of falls compared to simple walking alone [18, 26]. Moreover, the 30-s Sit-to-Stand (STS) test [48] was implemented to assess lower limb power and short-term muscle endurance. The participants commenced the test while seated in a chair and were then asked to stand up straight and sit down as often as possible within a time frame of 30 s. The count of times the participant fully stood up was evaluated.

### Statistical methods

Potential differences in demographics and test results between fallers and non-fallers were tested with an independent *t*-test for continuous variables and a chi-square test for dichotomous variables. Levene test was used to test the homogeneity of variances, and in case the homogeneity of variance assumption was not met, Welch *t*-test was interpreted.

To assess the assumption of linearity, the Box-Tidwell method was used. Leverage values (with the interpretation of Huber) and Cooks Distance were used to identify outliers. Outliers which were identified by both methods were then removed.

To analyze possible associations between all assessments and fall status, two main regression analyses were conducted: first, a binominal logistic regression analysis including all instrumented assessments and standardized geriatric assessments as independent variables and fall status as the dependent variable. Secondly, another binominal logistic regression analysis was conducted to evaluate the relative contribution of only relevant assessments for fall status. To determine these relevant assessments, single regression analyses were performed and those assessments that showed significant associations with fall status were then included again as independent variables in the second regression analysis (with fall status again as the dependent variable).

Additionally, to investigate a possible effect of age and cognitive functioning in the prediction of fall status, both regression analyses were repeated with age and MMSE as additional covariates.

Finally, a receiver operator curve (ROC) analysis was performed to analyze each test’s accuracy in classification and to directly compare AUC values of the instrumented assessments with the AUC value of the standardized geriatric assessments. Furthermore, the Youden Index was used to find the optimal cut-off values differentiating fallers from non-fallers.

All analyses were conducted with SPSS and significance level was set at *α* ≤ 0.05 (2-sided).

## Results

### Participants

One hundred and thirty-seven participants were included in this study, 38 categorized as fallers and 99 as non-fallers with fallers having experienced on average 2.5 ± 3.0 falls in the last 12 months. Table 1 provides an overview of the demographics of the included participants.

**Table 1:** Demographics of included participants

Variable	Total sample (*N*** = **137)			Fallers (*n*** = **38)			Non-fallers (*n*** = **99)			*p* value
Female, *n* (%)	83 (60.6%)			24 (63.2%)			59 (59.6%)			0.702
	Min.^a^	Max.^b^	Mean (SD^c^)	Min	Max	Mean (SD)	Min	Max	Mean (SD)	
Age	60	91	73.1 (7.3)	60	89	72.4 (7.1)	61	91	74.8 (7.5)	0.079
Body height (cm)	150	185	165.5 (8.1)	150	185	165.6 (8.0)	150	185	165 (8.4)	0.880
Body weight (kg)	48	110	72.9 (12.8)	48	110	73.3 (13.4)	52	98	71.8 (11.3)	0.545
Years of education	3	28	12.2 (4.7)	3	28	12.2 (4.8)	5	23	12.2 (4.5)	0.969
MMSE^d^ score	24	30	28.0 (1.7)	24	30	28.0 (1.7)	24	30	28.1 (1.7)	0.580

*Note.* ^a^*Min.* minimum, ^b^*Max.* maximum, ^c^*SD* standard deviation, ^d^*MMSE* mini mental state examination

### Descriptive statistics

Implausible values (probably due to data input errors) for the TUG and TUG-DT test (over 50 s) were recorded for one participant, which were naturally identified as outliers by both Cook’s distance and leverage values and thus excluded from further analyses. All descriptive test results categorized by fallers- and non-fallers can be found in Table 2.

**Table 2:** Test results non-fallers and fallers

Test	Fall status	Mean	SD	*p-*value	Cohen’s *d*	MD^a^	95% CI	
							Lower	Upper
Reaction Time Test (ms)	Total	1171.1	319.1					
	Non-fallers	1140.5	321.3	0.070	0.35	− 110.4	− 229.8	9.0
	Fallers	1250.9	303.2					
Go/No-Go (ms)	Total	972.0	170.0					
	Non-fallers	939.6	152.9	0.001*	0.72	− 116.7	− 184.2	− 49.2
	Fallers	1056.3	184.8					
Flexibility Test (ms)	Total	1856.9	978.8					
	Non-fallers	1822.0	1002.9	0.498	0.13	− 128.3	− 502.0	245.5
	Fallers	1950.3	917.8					
Sway Test (path length, mm)	Total	506.3	348.9					
	Non-fallers	463.2	164.7	0.168	0.41	− 155.6	− 379.4	68.2
	Fallers	618.8	674.2					
Sway Test (sway speed, mm/s)	Total	16.9	12.8					
	Fallers	15.4	5.5	0.165	0.41	− 5.2	− 10.0	− 0.4
	Non-fallers	20.7	22.4					
Coordinated Stability Test (deviation from ideal path, mm)	Total	2058.5	1792.0					
	Non-fallers	2044.1	1907.1	0.880	0.03	− 51.9	− 730.7	626.8
	Fallers	2096.1	1472.3					
Coordinated Stability (trace length, mm)	Total	2838.4	1813.0					
	Non-fallers	2806.0	1934.1	0.737	0.06	− 116.7	− 803.2	569.7
	Faller	2922.7	1470.8					
TUG^b^ (s)	Total	8.6	4.3					
	Non-fallers	8.0	4.1	0.014*	0.48	− 2.0	− 3.6	− 0.4
	Fallers	10.1	4.6					
TUG-DT^c^ (s)	Total	11.6	6.3					
	Non-fallers	11.0	5.7	0.123	0.35	− 2.2	− 5.0	0.6
	Fallers	13.2	7.7					
TUG-DT cost (%)	Total	− 34.3	30.8					
	Non-fallers	− 36.7	30.5	0.148	0.28	− 8.6	− 20.2	3.1
	Fallers	− 28.1	30.7					
STS^d^ Test (number of complete stand ups)	Total	12.7	4.9					
	Non-fallers	13.4	5.1	0.008*	0.51	2.4	0.6	4.2
	Fallers	11.0	3.9					

*Note.* ^a^*MD* mean difference between fallers and non-fallers, ^b^*TUG* Timed Up and Go, ^c^*TUG-DT* Timed Up and Go Dual Task, ^d^*STS* Sit-to-Stand, *p < 0.05

The independent *t*-tests showed significant differences between fallers and non-fallers with a medium-effect size in the Go/No-Go test, with mean reaction times 116.7 ms (95%-CI [− 116.7, − 49.2]) lower for the non-fallers (*t*(58) =  − 3.5, *p* = 0.001, Cohen’s *d* = 0.72). Regarding the standardized geriatric assessments, significant between-group differences and medium effect sizes were found for the TUG (mean difference: − 2.0, 95%-CI [− 3.6, − 0.4], *t*(134) =  − 2.5, *p* = 0.014, *d* = 0.48), and the STS Test (mean difference: 2.4, 95%-CI [0.6, 4.2], *t*(135) =  − 2.7, *p* = 0.008, *d* = 0.51).

### Regression model 1: binominal logistic regression analysis with all assessments as independent variables

In the first regression model, all assessments listed in Table 2 were included in one binominal logistic regression analysis as independent variables with fall status as outcome variable. High collinearity was detected for TUG and TUG-DT which is why TUG-DT was excluded from this regression model.

This first logistic regression model was statistically significant, *χ*^2^ (10) = 29.35, *p* = 0.001, resulting in an acceptable amount of explained variance [27], as shown by Nagelkerke’s *R*^2^ = 0.285. Only one variable, Go/No-Go (inhibition), contributed significantly to predicting fall status (*p* = 0.005). All model coefficients, significance values, and odds can be found in Table 3.

**Table 3:** Regression model 1: logistic regression analysis with all assessments as independent variables and fall status as the dependent variable

		*B*^a^	Sig	Exp(*B*)^b^	95% C.I. for EXP(*B*)	
					Lower	Upper
	Reaction Time Test	− 0.001	0.311	0.999	0.996	1.001
	Go/No-Go	0.006	0.005*	1.006	1.002	1.010
	Flexibility Test	0.000	0.303	1.000	0.999	1.000
	Sway path length	− 0.143	0.328	0.867	0.651	1.154
	Sway speed	4.337	0.322	76.485	0.014	411,624.331
	Coordinated Stability–path deviation	− 0.103	0.657	0.902	0.573	1.420
	Coordinated Stability–trace length	0.103	0.657	1.108	0.704	1.744
	TUG^c^	− 0.027	0.690	0.973	0.853	1.111
	TUG DT cost^d^	0.012	0.187	1.012	0.994	1.030
	STS^e^	− 0.061	0.320	0.941	0.835	1.061
	Constant	− 89.12	0.641	0.000		

*Note.* ^a^*B* regression coefficient *B*, ^b^*Exp(B)* odds ratio, ^c^*TUG* Timed Up and Go, ^d^*TUG-DT* Timed Up and Go–dual-task cost, ^e^*STS* Sit-to-Stand, *p < 0.05

### Regression model 2: binominal logistic regression analysis with significantly associated assessments as independent variables

As described above, in a second binominal logistic regression model, only assessments significantly associated with fall status were included as independent variables. To determine these assessments, single regression analyses were performed separately for each. In these single regression analyses, only Go/No-Go (*p* < 0.001), TUG (*p* = 0.019), and STS (*p* = 0.011) showed significant associations with the fall status and were, therefore, included in the second regression model.

This second binominal logistic regression model was statistically significant, *χ*^2^ (3) = 12.21, *p* = 0.007; however, explained only a low amount of variance (Nagelkerke’s *R*^2^ = 0.124) [27]. Again, only Go/No-Go contributed significantly to predicting fall status (*p* = 0.024). All model coefficients, significance values, and odds can be found in Table 4.

**Table 4:** Regression model 2: logistic regression analysis with assessments significantly associated with fall status as independent variables and fall status as the dependent variable

	*B*^a^	Sig	Exp(*B*)^b^	95% C.I. for EXP(*B*)	
				Lower	Upper
STS^b^	− 0.031	0.552	0.969	0.874	1.075
TUG^d^	0.008	0.900	1.008	0.896	1.133
Go/No-Go	0.003	0.024*	1.003	1.000	1.006
Constant	− 4.086	0.021	0.017		

*Note.* ^a^*B* regression coefficient *B*, ^b^*Exp(B)* odds ratio, ^c^*STS* Sit-to-Stand, ^d^*TUG* Timed Up and Go, *p < 0.05

### Covariates

In both models, adding age and the MMSE score as covariates did not result in statistically significant changes in *χ*^2^ values (s. supplementary Tables 1 and 2) and, therefore, did not affect the fit of the models.

### AUC analysis

**Fig. 2:**
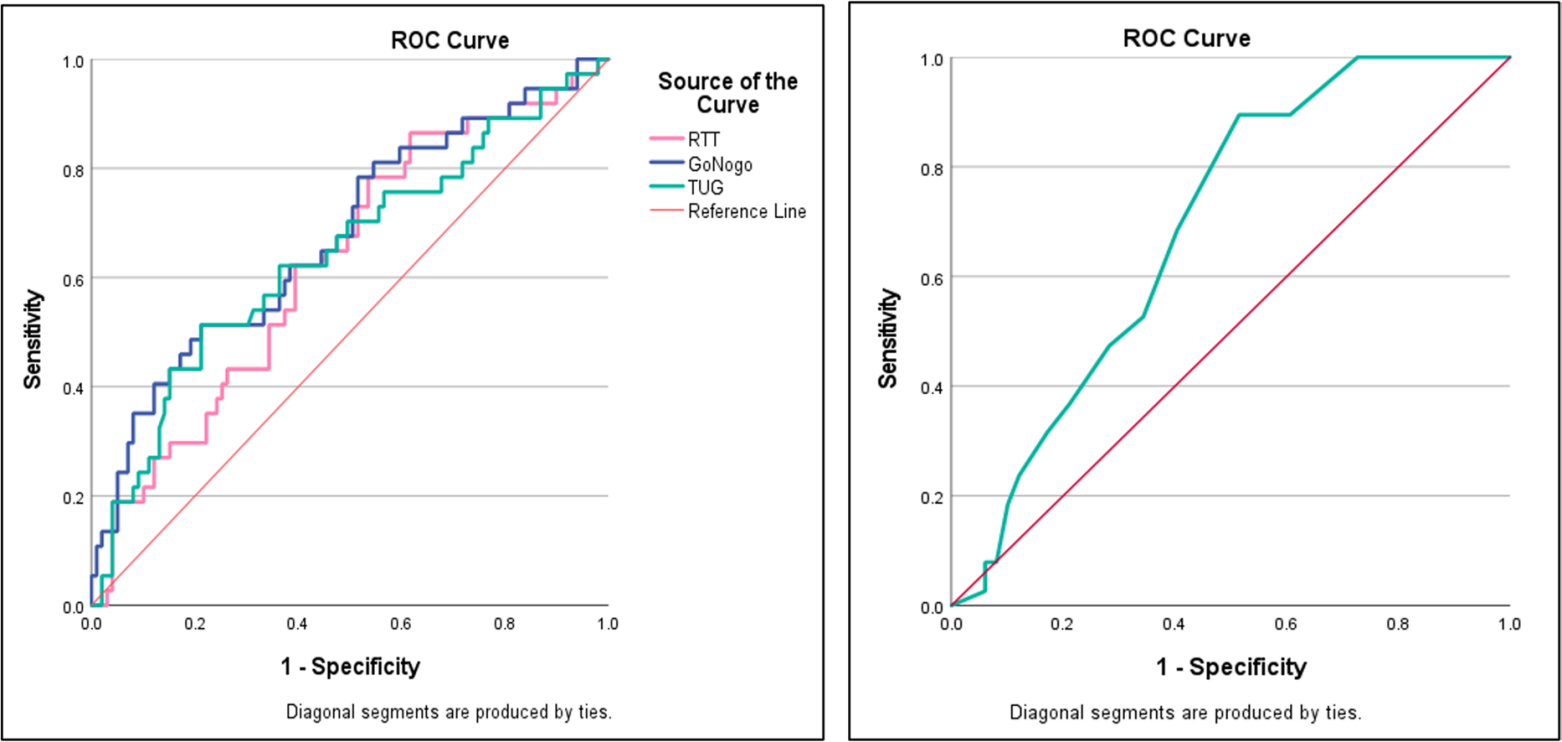
The ROC curves of the variables with significant AUC values

Significant AUC values were only found for the RTT (AUC = 0.628, *p* = 0.023), Go/No-Go (AUC = 0.673, *p* = 0.002), TUG (AUC = 0.642, *p* = 0.012), and STS (AUC = 0.690, *p* = 0.001). For the other variables, AUC values ranged between 0.541 (Coordinated Stability–path deviation, *p* = 0.465) and 0.579 (TUG DT cost, *p* = 0.161). Figure 2 shows the ROC curves of the variables with significant AUC values.

### Cut-off values

Cut-off values were determined for all variables in which fallers and non-fallers differed significantly (Table 2). Specifically, for the RTT, a reaction time of 994 ms was identified as a discriminative threshold, while for the Go/No-go task, this threshold was found to be 1074 ms. For the TUG test, a duration of 13.5 s emerged as a critical threshold for distinguishing fallers from non-fallers, and in the Sit-to-Stand (STS) test, 14.5 complete stand-ups.

## Discussion and implications

### Instrumented assessments with a cognitive component

The Go/No-Go test exhibited the strongest effect size in the direct performance comparisons of fallers and non-fallers and remained as a significant predictor in all regression models making it a potentially promising assessment tool for fall prediction. It is noteworthy, however, that although the AUC metric for Go/No-Go is the second largest of all assessments, its accuracy in classification was only moderate to acceptable [28, 29].

Still, this confirms the special role of inhibition for falls, as observed in other studies. For instance, Schoene et al. (2017) (p.723) [21] looked into the predictive ability of a similar step-based inhibition assessment and found that its effect on falls was “*direct and not mediated by processing speed, attention, and balance, further supporting the notion of iCSRT-RT [inhibitory choice reaction time] being an independent fall risk factor*.” Similarly, Mirelman et al. (2012) [19] found that among several computerized cognitive assessments, only response inhibition and attention were significantly associated with a future fall risk. This observation is not surprising, given the demands of daily activities and outdoor walking, where distractions frequently require a rapid response or the inhibition thereof.

This need for rapid response in order to avoid fall incidents might also explain the significant differences in RTT results between fallers and non-fallers, coupled with a statistically significant though poor accuracy in classification according to AUC analysis. However, the regression analyses did not yield statistically significant contributions from RTT, suggesting that, compared with inhibitory choice stepping reaction time (iCSRT), simple choice stepping reaction time (CSRT) holds less significance. This is consistent with previous research. For instance, Lord and Fitzpatrick (2001) [30] observed increased CSRT in older individuals prone to falls, yet subsequent studies such as Schoene et al. (2017) [21] who incorporated an inhibitory test component found that this component improved the predictive ability of CSRT.

Conversely, the cognitive flexibility test failed to differentiate fallers from non-fallers. Pieruccini-Faria (2019) [31] identified concept formation (a sub-component of cognitive flexibility) as a predictive factor for falls and also a confounding variable in the association between balance and falls. However, they also found that this confounding effect was more pronounced in individuals with poor balance, and the overall cognitive flexibility score did not emerge as a significant predictor for falls. These findings could explain the outcomes of our study in which participants exhibited a rather good balance and in which cognitive flexibility assessment was based just on a reaction time score.

### Instrumented assessments without a cognitive component

The instrumented assessments without a cognitive component (Coordinated Stability Test and the Sway Test) demonstrated a poor discriminatory ability in all statistical analyses, which is not in line with previous research showing strong associations between poor balance and an elevated risk of falls [31]. Our findings deviate, either entirely [32–34] or at least partly [30, 35], from previous research on fall risk factors.

For instance, similar to our study, Lord et al. (2001) [30] did not discover any significant differences in sway test measures between fallers and non-fallers. However, they did find worse performance in a Coordinated Stability Test in fallers. Noteworthy, the predictive ability of this Coordinated Stability Test turned out to be weaker compared to CSRT, and both the Coordinated Stability Test and the Sway Test exhibited significant associations with CSRT in the study by Lord et al. (2001).

One explanation for the differing results of our study regarding the discriminative ability of the balance assessments could be that, as described above, the balance ability of both fallers and non-fallers was high, for instance higher than that of participants in a prior usability study using the same Dividat Senso assessments [25] which is why a ceiling effect might have occurred. This in turn would explain why, in the current study population, balance is a non-determining factor in terms of fall risk. Findings by Johansson (2017) [33] supported this idea since they found a non-linear relationship between postural sway length and number of falls with a significantly greater fall frequency in the fifth quintile of sway length. Furthermore, the disparities from other studies might also be attributed to the slightly lower mean age in the present study, as age has exhibited strong negative correlations with balance ability and positive associations with the interplay of sensory, motor, and cognitive functions [31, 36]. Finally, as Zhou et al. (2017) [35] pointed out, standing postural sway is complex, as it depends on various inputs (e.g., somatosensory, visual, vestibular). Accordingly, they found that traditional postural sway metrics, such as those applied in our study, did not differ between fallers and non-fallers, whereas measures of CoP entropy—non-linear time-series analytical techniques—were able to predict falls. This was confirmed by a previous retrospective study revealing a stronger discriminative ability of such temporal dynamics as compared to traditional postural measures analyzing spatial dynamics of balance [37].

In summary, our results suggest that especially in a rather high functioning population, balance might play a subordinate role in fall risk and that simple balance metrics are not sufficient. This emphasizes the importance of integrating a cognitive component in the instrumented assessments.

### Standardized geriatric assessments

The TUG test results differed significantly between fallers and non-fallers and the test exhibited significant (though weak) accuracy in classification. However, it did not contribute significantly in either of the two main regression models. Thus, the TUG showed some discriminative ability, but, overall, this ability appeared weaker compared to the Go/No-Go Test.

This limited discriminative ability of the TUG aligns with previous literature [38] asserting its usefulness in fall risk assessment primarily in more frail older populations [39]. This is further underscored when examining cut-off values detected in the present (13.5 s) and in previous studies. Although proposed cut-off values vary widely between studies [39], most commonly ≥ 11 s or even ≥ 12.34 were recommended [6, 13, 39–41]. In our study, however, the average test completion time (8.6 s) was below most thresholds defined in previous studies. Another explanation is provided by Chiu et al. (2003) [42] who found that the TUG test is highly sensitive in differentiating multiple-fallers from non-fallers, however, less sensitive in differentiating single-fallers from non-fallers. In our study, though, the majority experienced only a single fall.

As mentioned in the introduction, it must be considered that the instrumented TUG might be more reliable in predicting fall status. For instance, Ponti et al. (2017) [13] found that the pure completion time was not significantly different between fallers and non-fallers and reached an AUC value of 0.668, whereas the fusion of features extracted from accelerometer data resulted in a significant group difference and increased the discriminative ability to an AUC value of 0.84.

In summary, as Chiu points out, in a rather healthy population the “*low discriminative ability of the TUG might indicate that the task involved could not challenge the mobility and balance functions of older people enough to reveal their risk for falls*”  [42 (p.48)].

Due to multicollinearity with the TUG, the TUG-DT had to be excluded from the first main regression analysis. Therefore, this regression analysis was repeated with the TUG-DT as an independent variable and with the TUG excluded instead. Remarkably, TUG and TUG-DT were mutually interchangeable without concomitant alterations in regression model accuracy and amount of explained variance (*χ*^2^(10) = 29.21, *p* = 0.001; Nagelkerke’s *R*^2^ = 0.283) and with a similar non-significant (*p* = 0.914) relative contribution to predicting fall status, suggesting either a diminished discriminative capacity or a shared measurement of the same construct in our population. This observation contrasts a number of prior studies that underscore the predictive superiority of dual-task assessments over single-task conditions [26, 43]. However, according to a recent systematic review comparing the ability of dual-task versus single-task tests to predict falls, only half of the included studies could confirm this superiority of dual-task tests which is why no definitive conclusions can be drawn yet [44].

Overall, there is conflicting evidence regarding whether to consider TUG(-DT) as standard geriatric fall risk assessments in the first place. Nevertheless, owing to their prevalent usage in clinical settings and the absence of universally accepted method, we opted to compare the instrumented assessments to TUG and TUG-DT, guided in part by recommendations such as those by Ambrose et al. [45].

The STS test stood out in the AUC analysis, showing the highest accuracy in classification of all assessments. This accentuates its potential as an efficient clinical fall risk assessment and is in line with previous research [46]. One explanation might be that STS performance reflects various sensorimotor functions, balance, psychological processes, and transfer skills [47], all of which have been associated with falls.

### Limitations

The biggest limitation of this study is that falls were assessed retrospectively. A prospective or longitudinal analysis is imperative for future investigations. Additionally, data on falls was collected based on self-report, leading to potential (although low) risk of recall-bias. Finally, the standardized geriatric assessments examined in this study are not exhaustive. There are other established geriatric tests which are used for fall risk screening such as the aforementioned Berg Balance Scale [9] and the Tinetti Mobility Test [10].

## Conclusion

Each instrumented motor-cognitive assessment alone lacked acceptable accuracy in fall status classification, whereas all assessments together yield the best results in terms of distinguishing between fallers and non-fallers. This is in line with previous research stating that due to the complex nature of fall risk, there is no ideal assessment, but the use of multiple assessments is recommended [48]. Nevertheless, the fact that Go/No-Go is the only test reaching significance in all analyses indicates a superiority of instrumented assessments with a cognitive component. Thus, it demonstrates that particularly assessing inhibition could indeed provide an added value for fall risk assessment and enhance the predictive ability of TUG, TUG-DT, and STS alone.

However, it is imperative to acknowledge that the participants in this study were physically and cognitively high functioning and reported a low incidence of falls. Results regarding the relative contribution of instrumented assessments with and without cognitive component and standardized geriatric assessments for fall risk could differ greatly in frail populations with mobility and/or cognitive impairments.

Finally, a notable advantage of all instrumented assessments is their high precision (compared to non-instrumented assessments). Extracted parameters from the instrumented assessment of this study were not exhaustive. Future studies should attempt to compute more parameters that examine their utility in fall risk assessment.

## Supplementary Information

Below is the link to the electronic supplementary material.

Supplementary file1 (DOCX 23 KB)

## Data Availability

All data is publicly available on Zenodo (https://doi.org/10.5281/zenodo.10805891).
