# Supplementary material for: Discriminative ability of instrumented cognitive-motor assessments to distinguish fallers from non-fallers
Source: GeroScience. 2024 Aug 9;47(1):1139–50. doi: 10.1007/s11357-024-01313-x (PMC11872953; doi:10.1007/s11357-024-01313-x)
Supplement: Supplementary file 1 — Supplementary file1 (DOCX 23 KB) [file 11357_2024_1313_MOESM1_ESM.docx]

**Supplementary Material**

**Supplementary table 1** Regression model 1 with age and MMSE score as a covariates

|  | **B^a^** | **Sig.** | **Exp(B)^b^** | **95% C.I.for EXP(B)** | |
| --- | --- | --- | --- | --- | --- |
|  |  |  |  | **Lower** | **Upper** |
| Age | .02 | .68 | 1.02 | .94 | 1.09 |
| MMSE^c^ Score | -.08 | .57 | .92 | .69 | 1.22 |
| Reaction Time Test | -.001 | .34 | 1.00 | 1.00 | 1.00 |
| Go/No-go | .01 | .01* | 1.01 | 1.00 | 1.01 |
| Flexibility Test | .000 | .24 | 1.00 | 1.00 | 1.00 |
| Sway path length | -.138 | .33 | .87 | .66 | 1.15 |
| Sway speed | 4.20 | .32 | 66.44 | .016 | 268449.6 |
| Coordinated Stability – Path deviation | -.107 | .64 | .90 | .57 | 1.41 |
| Coordinated Stability  – Trace length | .107 | .64 | 1.11 | .71 | 1.75 |
| TUG^d^ | -.035 | .61 | .97 | .84 | 1.11 |
| TUG-DT cost^e^ | .014 | .15 | 1.01 | 1.00 | 1.03 |
| STS^f^ | -.062 | .32 | .94 | .83 | 1.06 |
| Constant | -91.62 | .63 | .00 |  |  |

*Note.* This model is statistically significant (χ²(12) = 29.898, p = .003, Nagelkerke’s *R*² = .289);

^a^ B: Regression coefficient B, ^b^ Exp(B): Odds Ratio, ^c^ MMSE: Mini Mental State Examination, ^d^ TUG: Timed Up and Go, ^e^ TUG-DT: Timed Up and Go – dual task cost, ^f^ STS: Sit-to-Stand

*p<.05

**Supplementary table 2** Regression model 2 with age and MMSE score as covariates

|  | | **B^a^** | **Sig.** | **Exp(B)^b^** | **95% C.I.for EXP(B)** | |
| --- | --- | --- | --- | --- | --- | --- |
|  |  |  |  |  | **Lower** | **Upper** |
|  | Age | .020 | .505 | 1.020 | .962 | 1.082 |
|  | MMSE^c^ Score | .103 | .403 | 1.109 | .870 | 1.413 |
|  | STS^d^ | -.026 | .623 | .974 | .878 | 1.081 |
|  | TUG^e^ | -.002 | .980 | .998 | .884 | 1.127 |
|  | Go/No-Go | .004 | .023* | 1.004 | 1.000 | 1.008 |
|  | Constant | -8.560 | 0.75 | .000 |  |  |

*Note.* This model is statistically significant (χ²(5) = 13.258, p = .021, Nagelkerke’s *R*² = .135);

^a^ B: Regression coefficient B, ^b^ Exp(B): Odds Ratio, ^c^ MMSE: Mini Mental State Examination, ^d^ STS: Sit-to-Stand, ^e^TUG: Timed Up and Go

*p<.05
